# A combined transcriptomic approach to identify candidates for an anti-tick vaccine blocking *B. afzelii* transmission

**DOI:** 10.1038/s41598-020-76268-y

**Published:** 2020-11-18

**Authors:** Jos J. A. Trentelman, Radek Sima, Nicolas Krezdorn, Julen Tomás-Cortázar, Diego Barriales, Katsuhisa Takumi, Joe M. Butler, Hein Sprong, Michelle J. Klouwens, Veronika Urbanova, Sazzad Mahmood, Peter Winter, Petr Kopacek, Juan Anguita, Ondrej Hajdusek, Joppe W. Hovius

**Affiliations:** 1 Center for Experimental and Molecular Medicine, Amsterdam Infection and Immunity, Amsterdam UMC, Location Academic Medical Center, University of Amsterdam, Amsterdam, The Netherlands; 2 Biology Centre, Institute of Parasitology, Czech Academy of Sciences, Ceske Budejovice, Czech Republic; 3 GenXPro GmbH, Frankfurt Innovation Center Biotechnology, Frankfurt am Main, Germany; 4 CIC bioGUNE-Basque Research & Technology Alliance, 48160 Derio, Spain; 5 National Institute for Public Health and the Environment (RIVM), Bilthoven, The Netherlands; 6 Faculty of Science, University of South Bohemia, Ceske Budejovice, Czech Republic; 7 Ikerbasque, Basque Foundation for Science, 48012 Bilbao, Spain

**Keywords:** Vaccines, Bacterial infection, Transcriptomics, Parasite host response, Parasite immune evasion, Vaccines

## Abstract

*Ixodes ricinus* is the vector for *Borrelia afzelii*, the predominant cause of Lyme borreliosis in Europe, whereas *Ixodes scapularis* is the vector for *Borrelia burgdorferi* in the USA. Transcription of several *I. scapularis* genes changes in the presence of *B. burgdorferi* and contributes to successful infection. To what extend *B. afzelii* influences gene expression in *I. ricinus* salivary glands is largely unknown. Therefore, we measured expression of uninfected vs. infected tick salivary gland genes during tick feeding using Massive Analysis of cDNA Ends (MACE) and RNAseq, quantifying 26.179 unique transcripts. While tick feeding was the main differentiator, *B. afzelii* infection significantly affected expression of hundreds of transcripts, including 465 transcripts after 24 h of tick feeding. Validation of the top-20 *B. afzelii*-upregulated transcripts at 24 h of tick feeding in ten biological genetic distinct replicates showed that expression varied extensively. Three transcripts could be validated, a basic tail protein, a lipocalin and an ixodegrin, and might be involved in *B. afzelii* transmission. However, vaccination with recombinant forms of these proteins only marginally altered *B. afzelii* infection in *I. ricinus*-challenged mice for one of the proteins. Collectively, our data show that identification of tick salivary genes upregulated in the presence of pathogens could serve to identify potential pathogen-blocking vaccine candidates.

## Introduction

*Ixodes* ticks are small parasitic arthropods that feed on the blood of vertebrate hosts. They are three host-ticks; their lifecycle consists of four life stages, egg, larva, nymph and adult, where the latter three each parasitizes different hosts. Ticks needs to feed on blood of their hosts to obtain the nutrients and energy to develop into their next life stage or for successful reproduction. They do so by penetrating the skin of their host with their hypostome and, depending on the life stage, stay attached for 3–10 days to complete their blood meal. This feeding behavior presents a large window of opportunity for tick-borne pathogens to be transmitted to the host. Ticks are therefore only second to mosquitoes as the most important arthropod vectors for human disease. In contrast to the USA where *Ixodes scapularis* is the tick species most notorious for human disease^1^, in Europe, *Ixodes ricinus* is the tick that most affects human health^2^. *I. ricinus* is a vector for viruses, bacteria and protozoan parasites, and as such can cause a wide range of diseases, including tick-borne encephalitis, relapsing fever, anaplasmosis, babesiosis and most notably Lyme borreliosis.

Lyme borreliosis, also referred to as Lyme disease, is the most prevalent *I. ricinus*-borne disease; in Europe alone, over 65,000 cases of Lyme borreliosis are reported every year and some expect it to be 2–3 times higher due to underreporting^3^. Lyme borreliosis is caused by bacteria belonging to *Borrelia burgdorferi* sensu lato (s.l.) group and in Europe, *Borrelia afzelii* has the highest incidence rate. In humans, it is associated with (chronic) cutaneous manifestations of Lyme borreliosis^4^. *B. afzelii* is acquired by the larval tick during its first blood meal, it can survive in the tick to later life stages and can be transmitted with each following blood meal. However, given their smaller size nymphal ticks are less easy to be identified (visually and sensationally) than adult ticks and they are therefore considered to be the most clinically relevant life stage with regard to human disease^5^. It is commonly accepted that *B. burgdorferi* s.l. transmission starts approximately 16–36 h after attachment of the tick, transmission of *B. afzelii* starts earlier than *B. burgdorferi* sensu stricto (s.s.)^6,7^*.* In spite of this, it has been shown in a mouse model that *B. afzelii-*infected ticks need to feed for longer than 24 h to establish infection. *B. afzelii* is presumably transmitted through the saliva of the feeding tick, although alternative routes of infection have been proposed^8^.

The saliva of the tick is crucial for the long period of attachment and the successful completion of the blood meal. It contains proteins that interfere with host defense mechanisms through for instance immunosuppressive, anticomplement or antihemostatic roles. Indeed, animals repeatedly infested with ticks have antibodies against tick saliva and display so-called tick-immunity; ticks are less able to feed and/or are rejected^9–11^. As the host defense mechanisms are also essential to prevent and contain infection, these tick salivary gland proteins (TSGPs) greatly increase the odds of successful infection of the vertebrate host by *B. burgdorferi* s.l.-infected ticks, as it has been shown most notably for *I. scapularis*^12–16^. As a consequence, anti-tick immunity also protects against *B. burgdorferi* s.l. infection via tick bites and it has been shown that this anti-tick immunity can be transferred by serum^17–20^. These observations show the potential of anti-tick vaccines, by targeting tick proteins, specifically TSGPs, one could prevent tick feeding and/or pathogen transmission. Neutralization of specific TSGPs by antibodies indeed reduced *B. burgdorferi* s.s. infection *in vivo*^18,19^. As with all biological processes, the expression, translation and secretion of TSGPs is a dynamic process. The expression of these TSGPs is highly upregulated during the tick feeding process^21–24^, but it is also known that infection with *B. burgdorferi* s.s. induces alterations in gene expression that contribute to the successful infection of the host^13–15,25–29^. Based on their properties, TSGPs can be divided in large multi-gene families that have distinct functionalities, as reviewed before^30^. These multi-gene families are thought to be the result of gene duplication early in evolution^31^.

We used a combined transcriptomic approach to gain insight into the transcriptional changes within the salivary glands of *I. ricinus* during the complex interplay between the tick, the host and the pathogen. The respective strengths of both Massive Analysis of cDNA Ends (MACE) and RNAseq were combined to identify tick transcripts and subsequent processes influenced by *B. afzelii.* Gene expression in salivary glands of *I. ricinus* nymphs in different stages of feeding (unfed, 24 h and fully fed) and in different states of infection with regards to *B. afzelii (*infected and uninfected) were analyzed and characterized to identify specific TSGPs upregulated in *B. afzelii-*infected salivary glands. TSGPs upregulated in *B. afzelii-*infected salivary glands and those biologically validated were tested as *B. afzelii*-blocking anti-tick vaccines.

## Results

### RNA sequencing

In order to obtain long sequences to serve as our own framework for the annotation of the ensuing MACE analyses, RNA was prepared simultaneously for the construction of both the MACE and RNA sequencing libraries. Salivary gland and whole body RNA was isolated from *B. afzelii* CB43-infected *I. ricinus* nymphs and uninfected *I. ricinus* nymphs from the same parental lineage fed for 0, 24 or 72 h. RNA was pooled for all time points of *B. afzelii-*infected salivary glands, uninfected salivary glands, *B. afzelii-*infected whole body and uninfected whole body tick samples to obtain four cDNA libraries for RNAseq. The resulting cDNA libraries were used for paired-end sequencing and resulted in a total of 329,111,102 reads (Table 1) to be used for analysis, after elimination of duplicates and quality trimming. From these reads, 32,897 high quality contigs could be assembled. These formed our Master Reference exome (Master Reference) for the MACE analyses and represent an unprecedented source of *I. ricinus* sequence information.

**Table 1:** Summary of RNA sequencing reads after cleaning.

	R1 Reads	R2 Reads	Total Reads
*Uninfected I. ricinus nymphs*	37,960,637	37,960,637	75,921,274
*B. afzelii-infected I. ricinus nymphs*	40,784,777	40,784,777	81,569,554
*B. afzelii-infected I. ricinus whole body*	44,465,827	44,465,827	88,931,654
*Uninfected I. ricinus whole body*	41,339,909	41,339,909	82,688,620
Total	164,555,551	164,555,551	329,111,102

### MACE analysis

MACE was chosen as a quantitative tool as sequencing of the polyA captured cDNA molecules will result in one short read per molecule. In contrast, with RNAseq, one cDNA molecule will result in multiple reads. As such, MACE is excellent for detailed quantification of gene expression and has proven to be able to identify even low expressed genes^32,33^. RNA from salivary glands extracted at each time point (unfed, 24 h fed and fully fed) of *B. afzelii* CB43-infected (ISG) or uninfected (NISG) were used to prepare a total of six cDNA libraries for MACE. A total of 74.651.134 sequencing reads were processed (Table 2) and mapped against our Master Reference or assembled de novo and subsequently annotated against SwissProt, Trembl and the NCBI nucleotide database. Annotation resulted in the identification of a total of 93,096 unique transcripts of which on average 250–500 bp were covered by the MACE reads. We focused on transcripts that had more than one normalized read (read per million reads) in at least one of the MACE libraries to reduce the background signals. As a result, the number of transcripts used for further analysis was 26,179 unique transcripts.

**Table 2:** Summary of MACE reads after cleaning.

Library	Reads
ISG0h	12,954,933
ISG24h	15,916,749
ISGFF	12,701,261
NISG0h	12,449,936
NISG24h	9,553,944
NISGFF	11,074,311
Total	74,651,134

### Differential gene expression

**Figure 1:**
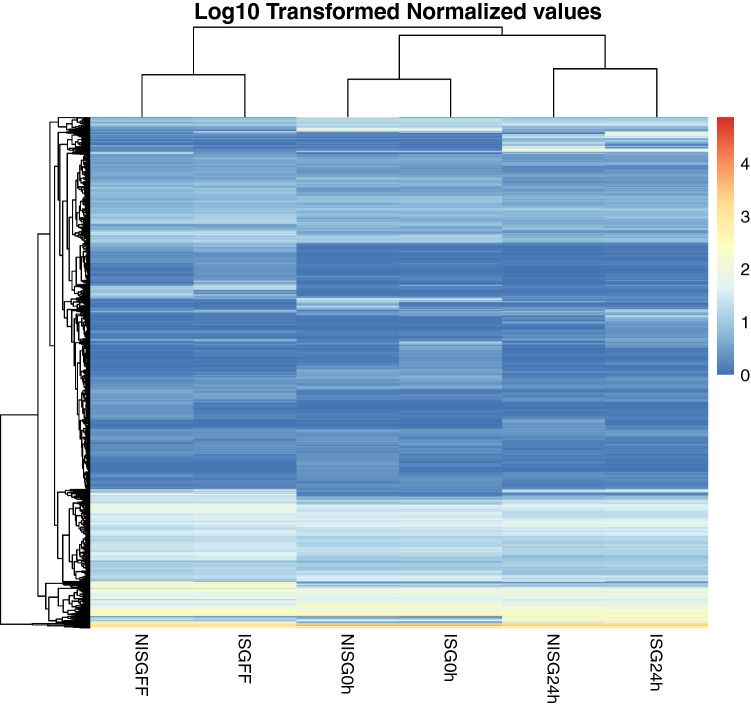
Unsupervised hierarchial cluster analyses of gene expression. Heatmap of log 10 transformed normalized reads illustrating gene expression of nymphal *I. ricinus* uninfected salivary glands (NISG) and *B. afzelii-*infected salivary glands (ISG) that were unfed (0 h), fed for 24 h (24 h) or fully engorged (FF). Each condition is represented in a single column. Gene expression is illustrated by color code, the color scale ranges from blue for low normalized reads to red for very high normalized reads.

**Figure 2:**
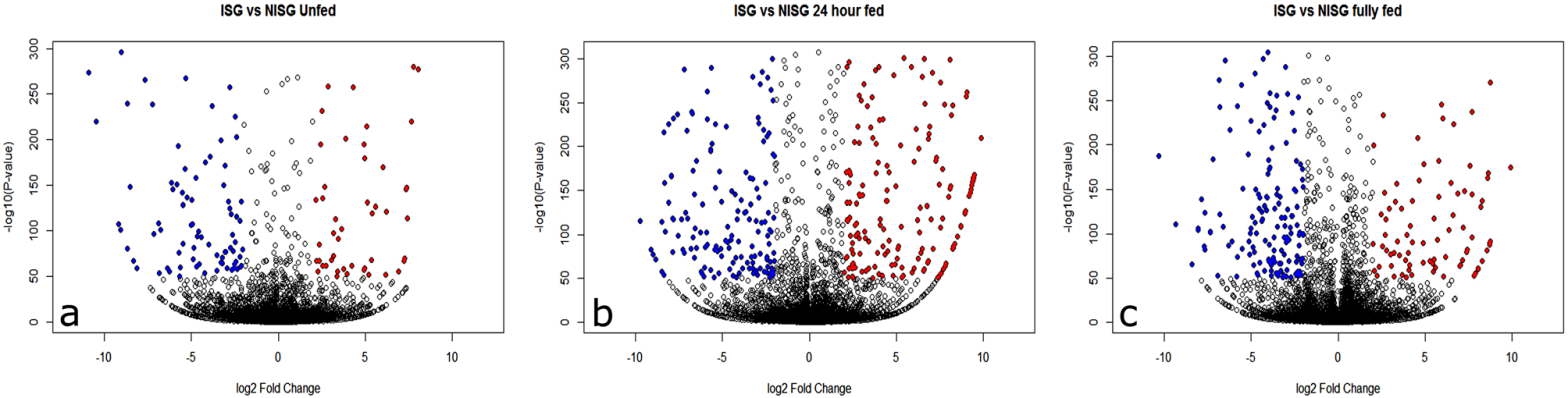
Volcano plot of comparison in salivary gland gene expression upon *B. afzelii* infection. (**a**) Relative gene expression in ISG vs NISG at 0 h. (**b**) Relative gene expression in ISG vs NISG at 24 h. (**c**) Relative gene expression in fully fed ISG vs NISG. Red dots are significantly upregulated genes, blue dots are significantly down regulated genes (log_2_fold < − 2 or > 2 and the corresponding *p* value < 1^–50^).

**Figure 3:**
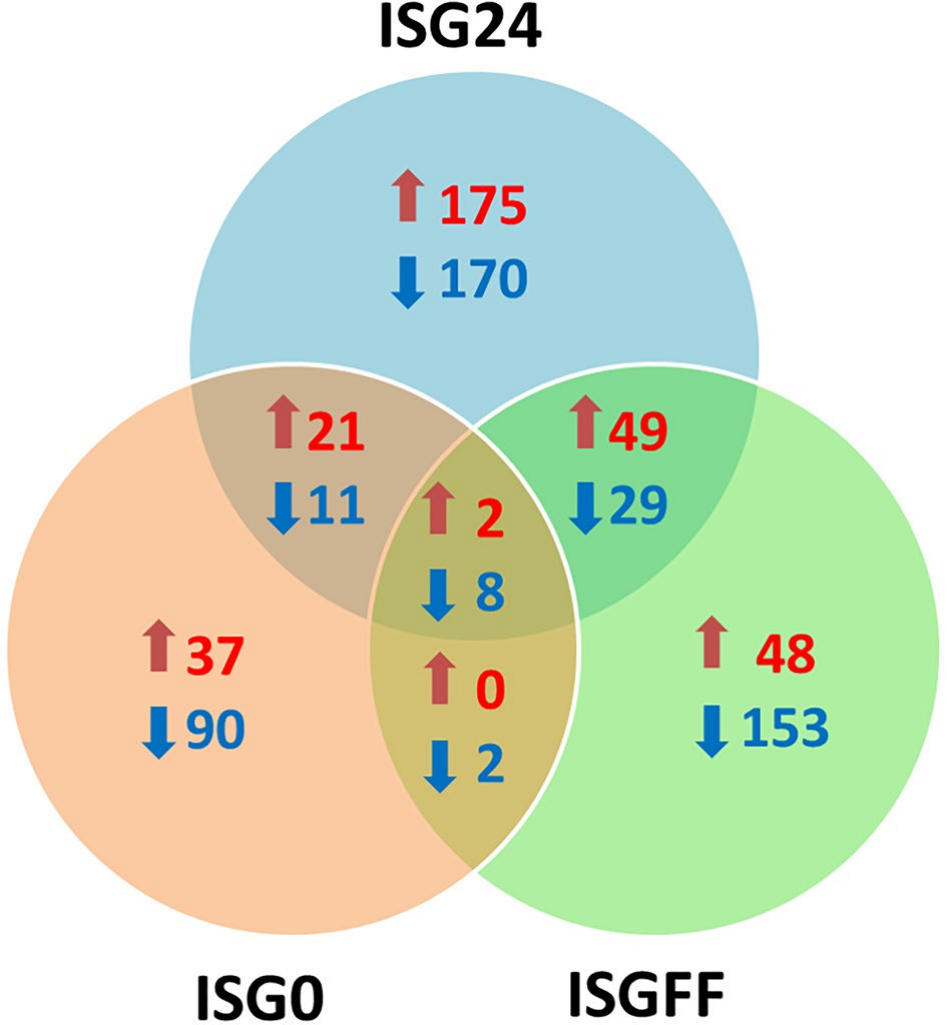
VENN diagram depicting differentially expressed tick salivary transcripts upon *B. afzelii infection* and their behaviour through time. Transcripts that are differentially up-regulated (> 2log2fold change in normalized reads, *p* < 1e−50) are depicted in red. Transcripts that are differentially down-regulated (> − 2log2fold change in normalized reads, *p* < 1e−50) are depicted in blue.

An unsupervised hierarchical clustering of all transcripts was performed (Fig. 1). From this analysis, it becomes clear that salivary gland gene expression was mostly affected by the stage of the feeding process. Differences in gene expression were most pronounced between early time points, 0 and 24 h fed, versus 72 h fed tick salivary glands (1790 and 1665 differentially expressed transcripts, respectively). Transcripts were considered to be differentially expressed when the change in gene expression was 4 times lower or 4 times higher, the corresponding *p* value < 1^–50^. Although gene expression was largely driven by the feeding status of the ticks, *B. afzelii* infection also altered gene expression (Fig. 2); in *B. afzelii*-infected unfed salivary glands (ISG0), 60 transcripts were upregulated and 110 transcripts were downregulated. In 72 h-fed *B. afzelii*-infected salivary glands (ISGFF) 99 transcripts were upregulated, while 192 were downregulated. Interestingly, most transcripts were differentially expressed upon 24 h feeding in *B. afzelii*-infected salivary glands (ISG24); 247 transcripts were upregulated and 218 were downregulated. Only a fraction of the genes were upregulated or downregulated at all time points (Fig. 3). Overall, *B. afzelii* infection influenced the expression of 795 unique salivary gland transcripts (> 2log2fold change or < -2log2fold change, *p* < 1e^−50^); 332 unique transcripts were up-regulated in one or more time points, whereas 463 unique transcripts were down-regulated in one or more time points. Interestingly, most transcripts that were affected by infection in a single time point only, were differentially expressed at 24 h of tick feeding (345 genes; 175 upregulated, 170 downregulated).

Thus, although differential gene expression in *I. ricinus* salivary glands was mostly driven by tick feeding, *B. afzelii* also influenced gene expression in *I. ricinus* salivary glands, and mostly at 24 h of tick feeding.

### Characterization of *B. afzelii*-induced differentially expressed tick salivary gland genes

To provide more insight into the possible biological functions of the differentially expressed tick salivary gland transcripts, these were assigned to known tick protein families. To this end, the corresponding contigs were aligned (blastx) to contigs of a previously described *I. ricinus* bioproject^23^, in which genes were eloquently assigned to different families of tick proteins. Our contigs that had a match with contigs from the previously described bioproject, with an Expect value below 0.00001, were assigned to the respective tick protein family^23^. Using this strategy, 81% of the differentially expressed transcripts could be annotated to a tick protein family.

**Figure 4:**
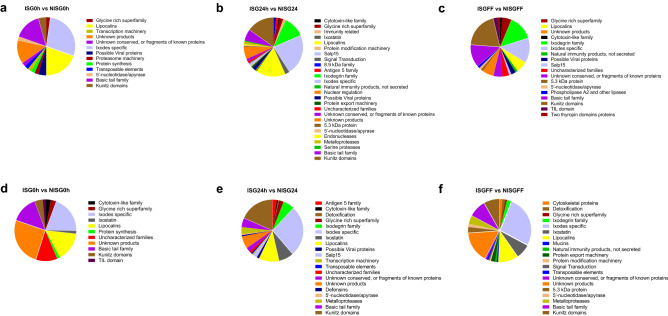
Distribution of differentially expressed transcripts over tick protein families for each time point. (**a**, **b**, **c**) Distribution of up-regulated transcripts (> 2 log2 fold change, *p* < 1^–50^) over tick protein families in *B. afzelii*-infected salivary glands of unfed (ISG0h vs NISG0h), 24 h (ISG24h vs NISG24h) and 72 h (ISGFF vs NISGFF) fed nymphs respectively. (**d**, **e**, **f**) Distribution of down-regulated transcripts (< − 2 log2 fold change, *p* < 1^–50^) over tick protein families in *B. afzelii*-infected salivary glands of unfed (ISG0h vs NISG0h), 24 h (ISG24h vs NISG24h) and 72 h (ISGFF vs NISGFF) fed nymphs respectively.

The functional annotation was limited to the main classes, only the classes of enzymes, antimicrobial peptides and protease inhibitor domains were divided into subclasses. Transcripts belonging to the glycine-rich superfamily, lipocalins, *Ixodes* specific family, and kunitz domain inhibitor family accounted for most of the transcripts upregulated by *B. afzelii* infection at any given time point (Fig. 4). Some tick protein families were only upregulated in ISG24h; most notably those related to immunity (1.46% of the upregulated transcripts at 24 h), ixostatin (2.44%), signal transduction related transcripts (0.49%), 8,9 kDa family (1.46%), antigen 5 family (1.46%), protein export machinery (0.98%), protein modification machinery (0.49%), metalloproteases (1.46%) and serine proteases (0.49%). Other families were upregulated at both ISG24h and ISGFF, those time points at which the tick is feeding and transmission of *B. afzelii* is taking place. Among these upregulated transcripts, members of the ixodegrin family (11.71% and 13.75% respectively) and Salp15 family (1.46% and 1.25% respectively) members were observed. In addition, although a few transcripts were upregulated in ISG0h (0.35% of upregulated transcripts), a marked increase of upregulated transcripts belonging to the kunitz domain inhibitor family were observed in ISG24h (15.12%) and ISGFF (20%) as well. Most of the transcripts upregulated at ISG24h belonged to the kunitz domain inhibitor (15.12%), ixodegrins (11.71%), Ixodes specific (22.44%) and lipocalin (16.10%) families.

Regarding the transcripts downregulated in infected salivary glands, the families affected at all time points were the glycine rich superfamily, *Ixodes* specific, lipocalins, kunitz domain families and transcripts that are considered as unknown products (with no homology to known sequences). Downregulated only in ISG24 were transcripts belonging to the Antigen 5 family (2.38% of the downregulated transcripts), Salp15 (2.38%), defensins (0.6%) and transcription machinery (0.6%).

Next to transcripts that were present in both uninfected and infected salivary glands, some transcripts could exclusively be detected in infected salivary glands, of which those only expressed in ISG24h are depicted in Supplemental Fig. 1. These transcripts were associated with the TIL—(Trypsin Inhibitor like cysteine rich) domain (4.17% of the transcripts only expressed in ISG24h compared to NISG24h), lipocalin (12.5%), Salp15 (4.17%), ixodegrin (16.67%), *Ixodes* specific (16.67%), ixostatin (8.33%) and, particularly, Kunitz domain families (33.33%).

Overall, *B. afzelii* was shown to affect *I. ricinus* salivary gland expression of transcripts encoding proteins belonging to multiple tick proteins families. Interestingly, we observed unique expression, as well as up-regulation and down-regulation, of transcripts within certain tick protein families, most notably *Ixodes* specific, lipocalins, basic tail protein, ixodegrin, kunitz domain inhibitor and ixostatin tick protein families.

### Selection of vaccine candidates; technical and biological validation

**Figure 5:**
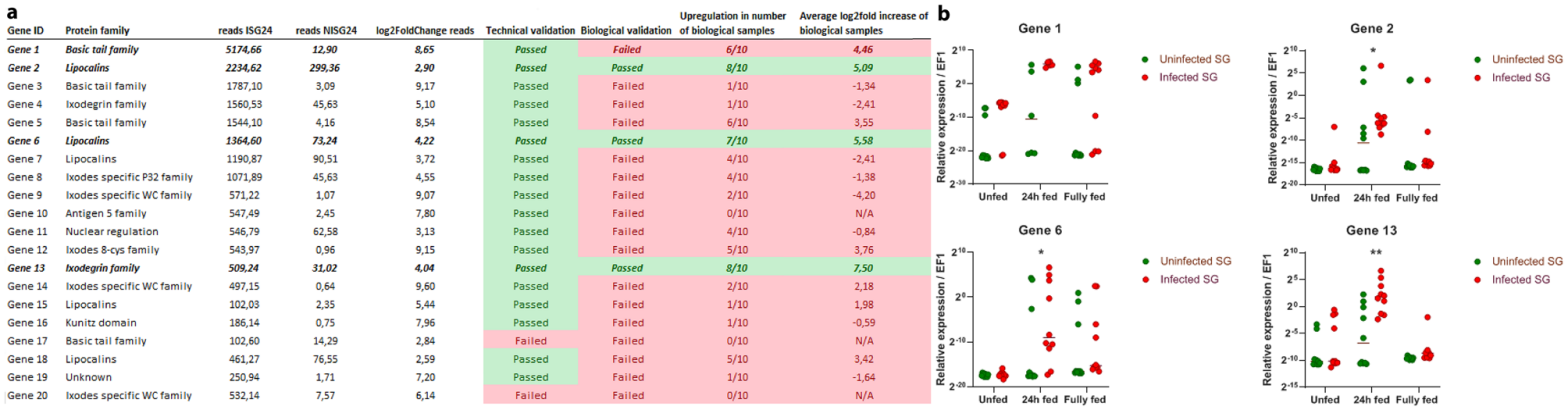
Technical and biological validation of top 20 vaccine candidates. (**a**) Top 20 genes highly upregulated in infected tick salivary glands at 24 h after onset feeding, (> 2 log2 fold change ISG24h vs NISG24h, *p* < 1 × 10^–50^) were considered potential *Borrelia* transmission blocking vaccine candidates. Top 20 was ranked based on expression levels in infected salivary glands at 24 h as determined by MACE. Of the 10 biologically distinct tick pools used for biological validation, the number of pools that showed upregulation of the respective transcript are indicated as well as the average log2fold difference in all 10 tick pools. (**b**) Gene expression profiles of biologically validated Genes 1, 2, 6 and 13 in the salivary glands of 10 biologically distinct tick pools as determined by RT-qPCR. Elongation factor 1 alpha was used as a reference gene. Lines indicated median expression values. Significantly upregulated transcripts are indicated by * (Friedman test paired analysis, Dunn’s multiple comparison *p* < 0. 05).

Tick salivary transcripts upregulated upon infection with *B. afzelii* might be important for transmission of *B. afzelii* and/or subsequent successful infection of the vertebrate host. Therefore, proteins encoded by transcripts that were highly upregulated in ISG24 were considered as potential candidates for a *Borrelia* transmission blocking vaccine. Significantly upregulated genes (> 2 log_2_, *p* < 1 × 10^–50^) were ranked based on expression levels determined by MACE and the 20 most abundantly expressed transcripts were selected for technical and biological validation. Primers were designed based on the nucleotide sequence identified by MACE and qRT-PCR was performed on the cDNA used for MACE (technical validation) or cDNA from tick pools derived from 10 genetically distinct ticks (biological validation). Technical validation showed that expression levels determined by qRT-PCR could confirm the MACE results for nearly all transcripts (Fig. 5), underscoring the robustness and accuracy of our approach. However, biological validation using cDNA from 10 genetically distinct tick pools showed marked variability in gene expression of the selected transcripts. Of the 20 selected transcripts, 3 genes were significantly upregulated in *B. afzelii*-infected tick salivary glands in most of the 10 genetically distinct tick pools; Gene 2, Gene 6 and Gene 13 (Fig. 5). In silico analysis showed that Gene 6 and Gene 2 are in fact highly similar; their sequence analysis showed 86% similarity at the amino acid level and Gene 6 appears to have a deletion compared to Gene 2. All 3 significantly upregulated transcripts encode a signal sequence and are likely to encode secreted proteins. Although not significantly upregulated at 24 h after the onset of feeding in the biological validation, Gene 1 was considered to be an interesting candidate. Gene 1 was only detected in 6 out of 10 tick pools, but in these tick pools Gene 1 was highly expressed upon infection at 24 h (Fig. 5). In addition, Gene 1 also encoded a signal sequence and showed a high degree of homology to basic tail proteins, although there were no conserved domains that might indicate possible functions of the encoded protein. Gene 2 and 6 were putative lipocalins and contain predicted histamine binding domains. Gene 13 was classified as a putative ixodegrin, containing a prokineticin domain and was part of the colipase-like superfamily. As Gene 2 and 6 were highly similar, Gene 1, 2 and 13 were selected for cloning and recombinant protein production in *E. coli*. For the selected targets the amino acid sequence, the predicted protein model, conserved domains and other characteristics are shown in Table 3.

**Table 3:**
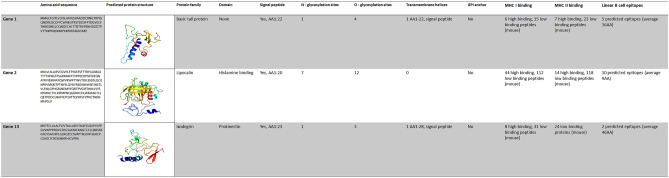
In silico analysis of validated transcripts selected for vaccination studies. Amino acid sequences encoded on the transcripts Gene 1, 2 and 13 as determined by the ExPASy Translate tool. Protein structures were predicted using Phyre2 web portal and although confidence in the predicted model was low for Gene 1 (32% of residues modelled at > 90% confidence, 55% of the sequence is predicted disordered), confidence in the predicted model was good for Gene 2 and 13 (73% and 67% of residues modelled at > 90% confidence respectively. Proteins sequences were subsequently scanned for domains with InterProScan. Signal peptide, O- and N-glycosylation sites were predicted based on amino acid sequence by SignalP 5.0 server, NetOGlyc 4.0 Server and NetNGlyc 1.0 server, respectively. HTHMM v2.0 server was used to predict transmembrane helices and GPI-SOM to predict GPI-anchor. MHC class I and II binding peptides were predicted using NetMHCpan-4.1 and NetMHCIIpan-4.0 and linear B cell epitopes with BepiPred Linear Epitope Prediction 2.0.

Columns colored in red are negative and those in blue are positive, while the others are neutral.

### Transmission and vaccination studies

Preliminary RNAi studies, with successful knock down of Gene1, 2, 6 and 13, in *B. afzelii*-infected nymphs fed on a small number of mice (n = 3), did not show a significant reduction of tick feeding or *B. afzelii* infection (Supplemental Fig. 2). This result indicated that the absence of transcripts by itself was not enough to affect *B. afzelii* transmission. We next focused on vaccination studies where antibody–antigen interactions and complexes can lead to multiple effector mechanism that can block transmission. To this end, mice were vaccinated with recombinant proteins of Gene 1, 2, 13 or a combination of these antigens and subsequently challenged with *B. afzelii*-infected nymphal ticks. Vaccination was shown to be successful; antigen-specific total IgG levels could be detected after vaccination (Supplemental Fig. 3), although antibody levels against recombinant Gene 2 were significantly lower as compared to the other antigens (Mann–Whitney test, *p* < 0.05). Vaccination with recombinant Gene 1 significantly reduced the number of infected mice tissues as determined by qPCR and although the number of mouse tissues infected as determined by culture was also lower, this effect was not significant nor was there a difference in the cumulative number of mice that were infected (Chi-square, *p* < 0.05) (Table 4). For all other experimental groups, including the cocktail vaccination, no significant differences were observed in the spirochetal loads of the tissues nor in the number of infected mice (Supplemental Fig. 3).

**Table 4:** Number of *Borrelia-*infected mice as determined for each organ.

	Skin^1^	Bladder^1^	Skin^2^	Bladder^2^	Heart^2^	Joint^2^	Cumulative
PBS	5/6	4/6	6/6	6/6	6/6	5/6	6/6
Recombinant Gene 1	3/6	3/6	2/6*	2/6*	2/6*	2/6*	5/6
Recombinant Gene 2	4/6	4/6	4/6	4/6	4/6	4/6	4/6
Recombinant Gene 13	4/6	4/6	4/6	4/6	4/6	5/6	5/6
Recombinant Gene 1 + Gene 2 + Gene 13	5/6	4/6	4/6	4/6	5/6	5/6	6/6

*Borrelia* infection as determined by culture^1^ or qPCR^2^ and shown as number of positive mice/total mice. Cumulative infection was calculated as the number of mice that were positive in at least one of the organs either by culture or qPCR. Significance was calculated compared to the PBS groups and significant differences are indicated by * (Chi-square, *p* = 0.04).

## Discussion

To our knowledge, this is the first time that the relationship between *B. afzelii* and nymphal *I. ricinus* on the total transcript level of salivary glands is studied. In the current study, two different gene quantification tools have been combined to provide an unprecedented insight into the transcriptome of *I. ricinus* salivary glands. RNAseq is a powerful technique to obtain accurate and qualitative sequence information of transcripts, but fragmentation of the RNA molecules and sequencing of all fragments could lead to a bias in the quantification of longer transcripts^34^. MACE, on the other hand, only targets sequences from the 3′ end of the sequence by capturing the RNA fragment containing the pol-A tail. As a result, sequence information might be partial (i.e. not providing sequence information of the whole gene sequence), but it provides a high resolution gene expression analysis, even revealing differential expression of low-abundant transcripts, which are beyond the scope of RNAseq or microarrays^35^. In addition, the TrueQuant method increases the reliability of quantification by eliminating PCR bias^36^. By combining RNAseq and MACE, the complete sequence information provided by RNAseq results in increased mapping accuracy of MACE reads, strengthening the highly accurate quantification by MACE. RNAseq analysis was performed using pooled RNA from nymphal *I. ricinus* salivary gland and whole bodies fed for different time points (0, 24 or fully fed), with or without *B. afzelii* infection, resulting in 32,897 high-quality contigs, which is similar to or higher than the number of transcripts reported by previous RNAseq projects^37–39^. MACE resulted in the quantification of 26.179 transcripts selected for further analysis. Technical validation by qRT-PCR using the MACE cDNA libraries, to determine the expression profiles of the 20 most abundantly expressed *B. afzelii*-induced *I. ricinus* salivary glands transcripts, corroborated the MACE expression profiles and clearly validated our findings.

As described previously, our results confirm that the feeding process greatly affected gene expression in tick salivary glands^37–39^. Although the feeding process is the main differentiator of gene expression, MACE analysis showed that the expression of hundreds of transcripts is significantly affected by *B. afzelii* infection. This could have multiple underlying mechanisms that are not mutually exclusive; firstly, the transcripts could be part of the tick immune response to *Borrelia* infection. Secondly, the expression could be altered by *Borrelia* infection to increase survival in the tick. Thirdly, the transcripts could be affected by *Borrelia* infection to increase transmission through saliva and infectivity in the mammalian host. Interestingly, only a few transcripts were upregulated in *B. afzelii-*infected salivary glands of unfed ticks. This fits the general assumption that there are few to none spirochete in the salivary glands at this time point as has been observed for *B. burgdorferi s.s.* as they are located in the midgut and still have to migrate to the salivary glands upon onset of feeding^40^. In addition, the expression of hardly any transcript is affected in all three time points (0, 24 and FF), making it unlikely that the identified upregulated transcripts are involved in the tick immune response against *B. afzelii.* Most of the *B. afzelii*-induced differentially expressed transcripts were observed 24 h after onset of tick feeding. This coincides with the time point that *B. burgdorferi* s.l. is thought to have found its way into the tick saliva and starts to be transmitted to the host^7^. Indeed, transmission experiments using the same experimental model that we have previously used, has shown that removal of *B. afzelii-*infected ticks after 24 h of tick feeding blocks successful infection of the host^41–43^.

As described above, it is known that certain *I. scapularis* TSGPs are upregulated upon *B. burgdorferi* infection and that some of these proteins are to be beneficial for the transmission success of the spirochete. However, this is the first study to investigate whether and to what extend *B. afzelii* influences gene expression in the salivary glands of nymphal *I. ricinus* ticks. With 465 transcripts differentially expressed at 24 h after onset feeding, the MACE analysis indicates that *B. afzelii* infection has an extensive effect on gene expression. The majority of transcripts upregulated in *Borrelia-*infected SG at 24 h belong to the kunitz domain inhibitor, ixodegrins, *Ixodes* specific and lipocalin protein families. Kunitz domain inhibitors are one of the largest families of secreted salivary gland proteins. These proteins have one or multiple kunitz domains that inhibit activity of specific proteases, most of which are involved in the coagulation pathway^30,44^. Ixodegrins are cysteine rich proteins that have a RGD or KGD domain, which can bind to integrins; transmembrane receptors that mediate cell–cell and cell–extracellular matrix interactions and as such can have multiple functions. Ixodegrins can block the interaction of integrins with their other ligands and block downstream processes. For instance, binding of ixodegrins to αIIbβ3 of activated platelets, prevents fibrinogen–platelet interaction and platelet aggregation^45^. Integrins, as they are transmembrane receptors, are also involved in immunity. For instance, macrophage 1 antigen, more recently known as complement receptor 3 (CR3), is an integrin present on polymorphonuclear leukocytes and binds fibrinogen which leads to macrophage adhesion and activation. Interestingly, CR3 interacts with uPAR, which has been shown to be important for the clearance of *Borrelia* and CR3 also binds *Borrelia* directly to polymorphonuclear leukocytes^28,46–49^. As the ligands of most ixodegrins are currently unknown, it might be possible that some could protect *Borrelia* from the host’s immune response. Ixodes specific protein family comprises several smaller families and only some members of the Isac protein subfamily have been characterized; these tick proteins interfere with the complement cascade^50–52^. The complement cascade is an important line of defense against *B. burgdorferi* s.l.*.* Although sensitivity for complement-mediated killing varies between *B. burgdorferi* s.l. genospecies and *B. afzelii* is particular complement resistant, complement leads to opsonophagocytosis of *B. burgdorferi* by immune cells and in antibody-dependent complement-mediated killing^53,54^. Thus, it is possible that the proteins upregulated in *B. afzelii*-infected *I. ricinus* salivary glands at 24 h after feeding facilitate both *B. afzelii* transmission from the tick to the host or successful infection of the host. However, as transcripts belonging to the same protein family are both upregulated and downregulated, the characterization of the majority of proteins in each protein family is poor or non-existent, and the same TSGP can exert multiple functions, it is difficult to appreciate the exact biological role of the different families of tick proteins in *B. afzelii* transmission or infection.

One of the aims of this study was to identify possible pathogen transmission blocking anti-tick vaccine targets. Previous studies have shown that antibodies against *I. scapularis* TSGPs not only interfere with tick feeding, but antibodies induced after 24 h of tick feeding could also partially protect against *B. burgdorferi* infection^11,17–19,55^. In search for potential vaccine targets to block *B. afzelii* transmission by *I. ricinus*, the 20 most abundantly expressed transcripts upregulated in *B. afzelii*-infected *I. ricinus* salivary glands at 24 h after onset feeding were validated in 10 biological and genetically distinct replicates. Three transcripts—encoding 2 unique proteins—were significantly upregulated in *B. afzelii-*infected salivary glands across the 10 biological samples; Gene 2, Gene 6 and Gene 13. Despite the fact that we, in line with previously published tick transcriptome studies^21,23,24^, pooled salivary glands of hundreds of ticks to obtain enough RNA for both RNAseq and MACE, we were only able to biologically validate three out of the 20 selected abundantly-expressed *I. ricinus* transcripts. This suggests that there is substantial biological variation, either in transcript sequence or expression and underscores that it is critical to consider this variation, especially when selecting vaccine candidates. Ideal vaccine candidates are highly conserved and expressed in multiple biological replicates, which is the case for the three selected transcripts. Gene 2 and 6 proved to encode the same protein, a putative lipocalin with predicted histamine binding domains. Lipocalins are one of the largest and most diverse protein families in ticks. Despite their diversity in amino acid sequence, they all have a barrel structure that creates a fold and facilitates the binding of hydrophobic ligands. The targets of lipocalins are as diverse as the protein family itself; lipocalins can target inflammation, acquired immunity and the complement system. As Gene 2 and 6 have histamine binding domains, they appear to belong to the first category. Histamine release by host cells induce inflammation at the tick bite site; hence, Gene 2 and 6 could inhibit inflammation by binding histamine. Gene 13 is characterized as a putative Ixodegrin, has a prokineticin domain and is part of the colipase-like superfamily. As described above, Ixodegrins are cysteine-rich proteins and although the function for most of these proteins is unknown, there are some that act as antiplatelet inhibitors and they might affect innate immunity^30,46,47,56,57^. In addition, although overall not significantly upregulated in *B. afzelii*-infected salivary glands at 24 h, Gene 1 was highly expressed in the tick pools in which the transcript could be detected and therefore evaluated as a vaccine candidate. In silico analysis showed that Gene 1 showed a high degree of homology to basic tail proteins and has a very basic carboxy terminus or tail, which is one of the key features of this protein family. It is thought that the basic tail might help binding to anionic phospholipids expressed at the surface of activated platelets and mast cells and can interfere in the functioning of the subsequent host processes^30,58^. Indeed, several TSGPs belonging to the basic tail protein family have been described to interfere with complement or coagulation (TSLPI, Salp14, Ixonnexin and Salp9pac)^15,16,44,59–61^. These processes have been proven to be important for tick feeding and *B. burgdorferi* survival in the host^15,16,61^. Although there are no conserved domains to directly pinpoint possible effector functions of Gene 1, the presence of a basic tail and upregulation in *B. afzelii-*infected salivary glands indicate that this TSGP could facilitate *B. afzelii* transmission or survival in the mammalian host. Thus, in silico analysis indicated that the three selected targets could very well be involved in the manipulation of the host defense mechanisms that are essential for tick feeding and/or survival of *B. afzelii* in the host. However, a preliminary RNAi experiment, in which the four validated transcripts encoding the three targets were successfully silenced, did not reveal an essential role for the identified TSGPs in tick feeding or *B. afzelii* transmission.

In line with these findings, vaccination with recombinant forms of Gene 2 or 13 did not reduce tick feeding nor *B. afzelii* transmission to the host after challenge with *B. afzelii-*infected *I. ricinus* nymphs, compared to control mice. In silico analysis showed that all antigens are predicted to have peptides that can bind to MHC class I and class II, and are linear epitopes for B cells. Vaccination indeed did induce high antibody titers for recombinant Gene 13, confirming immunogenicity. For recombinant Gene 2, antibody levels are relatively low despite predicted immunogenicity and although the purified antigen seem to contain *E. coli* residue that could interfere with the immune response, we consider it unlikely that these trace amounts of contaminants have interfered with the antigen specific immune response (Supplemental Fig. 3). It is therefore unclear what explains the modest antibody titers. Interestingly, vaccination with recombinant Gene 1 significantly induced a robust antibody response and reduced the number of infected tissues in mice as determined by qPCR. However, vaccination with Gene 1 did not protect against infection; the cumulative number of infected mice as determined by qPCR and culture was similar for recombinant Gene 1 vaccinated and control animals. This modest effect was not observed in mice vaccinated with all three antigens. This might be explained by interference of the other two antigens with the immune response against recombinant Gene 1 upon vaccination or tick-challenge. In general, other vaccination platforms or different ways of producing the tick antigens as recombinant proteins might lead to improved vaccine efficacy. Indeed, a recent publication showed the importance of glycosylation of tick saliva proteins in tick immunity against *I. scapularis*^9^. Therefore, one could argue that the fact that we produced the selected tick antigens in an *E. coli* expression system, and the resulting absence of posttranslational modifications such as glycosylation, are responsible for the low observed vaccine efficacy. Whether vaccination with Gene 1 produced in an Eukaryotic expression system would increase vaccine efficiency remains to be investigated.

To conclude, in this study, using two independent next generation sequencing techniques, we clearly show that *B. afzelii* affects *I. ricinus* salivary glands gene expression during tick feeding, and that the uniquely expressed, as well as up- and downregulated tick transcripts upon *B. afzelii* infection encode proteins belonging to the same tick protein families. Four transcripts encoding three different proteins were shown to be robustly upregulated in *B. afzelii*-infected *I. ricinus* salivary glands at 24 h. Of these three proteins, only recombinant Gene 1 altered *B. afzelii* infection when tested as a transmission blocking anti-tick vaccine in the current set-up and although it did not prevent infection, it could still be an interesting antigen for further optimization, for example as part of a multivalent vaccine or produced in a different expression system. In addition, future research could focus on determining the function of these proteins in either the tick or the host.

## Material and methods

### Infection of ticks with *Borrelia afzelii*, tick feeding and RNA extraction

*I. ricinus* ticks were obtained from the BC ASCR tick colony and were free of *Borrelia*, *Babesia*, and *Anaplasma,* as determined by PCR^62,63^. To obtain non-infected and *B. afzelii*-infected ticks, clean *I. ricinus* larvae—a mixture of the offspring from three individual adult females—were fed on naive or *B. afzelii* strain CB43 syringe-inoculated 6–8 weeks old C3H/HeN mice (Charles River Laboratories, Sulzfeld, Germany). Larvae were collected and allowed to molt to nymphs in a climate chamber with a humidity of about 95%, temperature 24 °C and day/night period set to 15/9 h. Infection rates for infected ticks were assessed by qPCR and ticks were used when infection rates were higher than 90%. Resulting non-infected and *B. afzelii*-infected ticks (4 to 6 weeks after molting) were fed for 0 h (220 nymphs per infection state, 440 total), 24 h (180 nymphs, 380 total) or to repletion (150, 300 total) on naive 6–8 weeks old C3H/HeN mice and dissected under a dissection microscope. Salivary glands were collected and total (small and large) RNA was extracted using a NucleoSpin miRNA kit (MACHEREY–NAGEL, Dürren, Germany) according to the manufacturer’s instructions and stored at − 80 °C until further use. All tick and animal experiments were approved by the BC ASCR animal ethical committee (Animal protection laws of the Czech Republic No. 246/1992 Sb., Ethics approval No. 79/2013). All experiments were performed in accordance with relevant guidelines and regulations.

### RNA sequencing

For RNA sequencing, we created four separate RNA-Seq libraries; infected salivary glands (RNA from 550 *B. afzelii*-infected nymphs at 0, 24 h and fully fed were pooled), uninfected salivary glands (RNA from 550 uninfected nymphs at 0, 24 h and fully fed were pooled), 45 *B. afzelii*-infected whole body fully fed nymphs and 45 uninfected whole body fully fed. The RNAseq libraries were generated using the “NEBNextUltra directional RNA-Seq” (NEB, Ispawich, USA) protocol, as described by the manufacturer and based on the method previously published^64^. In short, mRNA was captured from 5 μg of total RNA using Oligo dT(25) beads. The purified mRNA was randomly fragmented in a Zn^2+^ solution and first strand synthesis was performed using random hexamers. Second strand synthesis was performed using a dNTP mixture in which dTTP was exchanged with dUTP and P5-P7-Y-adapers were ligated. The second strand was eliminated prior to PCR using dUTPase. Subsequently, a PCR was performed using 14 cycles. The final products were analyzed on an Agilent 2100 Bioanalyzer (Agilent, Santa Clara, CA, USA) and product sizes ranged from 200 to 800 bp, with a major peak at 450 bp. Finally, the products were sequenced on an Illumina HiSeq2000 machine (Illumina, Inc., San Diego, CA, USA) using 2 × 100 bp. Overlapping sequencing reads were de novo assembled into GXP_Contigs with TrinityRNAseq (Version: v2.2.0^65^). Further assembly output refining resulted in 32,897 high quality Contigs used as a reference database. The obtained sequences were uploaded to GenBank (Bioproject PRJNA657487).

### MACE analysis

Essentially, MACE analysis was performed as previously described (Nold-Petry et al. Mueller et al.) using the GenXPro MACE kit (GenXPro, Frankfurt am Main, Germany) and according to the manufacturer’s protocol. Briefly, 1 µg of obtained large and small tick salivary gland RNA from 550 *B. afzelii* CB43-infected or 550 non-infected ticks fed for 0, 24 or approximately 72 h (fully fed) were subjected to an additional DNAse treatment to remove all DNA. Quality was assessed on an Agilent 2100 Bioanalyzer and no or only negligible degradation products were observed. Next, first and second strand cDNA synthesis was performed starting from biotinylated oligo dT primers. The cDNA was fragmented randomly by ultrasonication resulting in fragments with an average size of 300 bps as determined by an Agilent 2100 Bioanalyzer. The biotinylated 3′ cDNA ends were bound to a streptavidin matrix and all other fragments were eliminated through washing. To the unbound end of the fragments, a p5 “TrueQuant” sequencing adapter included in the MACE kit was ligated and a PCR was performed, using tailed Illumina p5 and p7 oligonucleotides as primers, in order to obtain a library of fragments suitable for Next Generation Sequencing on an Illumina Hiseq2000 machine. The Quality of the final library was determined using an Agilent 2100 bioanalyzer. Single end sequencing of the products produced the sequence-information of the 5′ side of the bound cDNA fragment. To remove PCR-bias, all duplicate reads detected by the in house TrueQuant technology were removed from the raw datasets. In addition, low quality sequence nucleotides and poly(A)-tails were clipped off using cutadapt^66^. The reads were thereafter aligned to different reference sequences using Novoalign (Novocraft Technologies, Selangor, Malaysia).The main reference for the Novoalign alignment was the outcome of the RNASeq de novo assembly, described in the RNASeq section. Additionally a de novo assembly of MACE sequences that could not be mapped to sequences from the Master Reference (RNASeq) using TrinityRNAseq (Version: v2.2.0 ^65^) was performed. Subsequently, the contigs of the assemblies, "Master Reference" and "noHitAssembly" were annotated further by BLASTX to first the SwissProt and hereafter Trembl database “Arachnida” proteins^67^. Additional blastn analyses were performed for all Contigs against all “Ixodes” mRNA sequences available at the NCBI database, nucleotide collection from GenBank (RefSeq, TPA and PDB), *Ixodes scapularis* genome (PRJNA314100), *Ixodes ricinus* genome (PRJNA270959) and against sequences from a previous published *I. ricinus* salivary gland transcriptome^23^ submitted to Genbank (PRJNA177622). The e-value threshold for BLASTX and BLASTN was 0.00001. Only uniquely mapped reads were accepted for quantification of the MACE tags. Finally, the expression was normalized and tested for differential gene expression between the different conditions using the DEGSeq R/Bioconductor package^67^. Only transcripts with at least 1 normalized read in one of the libraries were used for analysis (Supplemental file 1).

### Allocation of genes to tick protein families

For more functional insight, the transcripts were allocated to tick protein families based on sequence homology. In short, gene sequences of the Master Reference were aligned to proteins from a previous bioproject^39^ using blastx. Transcripts were considered to belong to a specific tick protein family if the e-value with their respective protein hit from the Bioproject Number PRJNA177622 was below 0.0001.

### Technical and biological validation

An aliquot of total RNA from each time point analyzed by MACE was used to make cDNAs (Transcriptor High Fidelity cDNA Synthesis Kit (Roche, Basel, Switzerland)) for qRT-PCR technical validation of the MACE results. For biological validations, *Borrelia afzelii*-infected (Infection rates were assessed by qPCR and ticks were used when infection rates were higher than 90%) and uninfected nymphal *I. ricinus* ticks derived from 10 distinct egg batches laid by adult female ticks collected from the wild, were fed on mice for different time points. RNA was isolated from the salivary glands and subsequent cDNA was prepared for the individual time points. Then, gene-specific primers appropriate for unambiguous PCR confirmation of gene expression in *Borrelia*-infected nymphs at the time interval 24 h and the genes upregulated by feeding, were designed using Primer3 software (Supplemental Table 1). qRT-PCR was used to evaluate expression of the selected genes in technical and biological samples.

### In silico analysis

The encoded protein sequence for Gene 1, 2 and 13 were determined from the transcripts nucleotide sequences using the ExPASy translate tool^68^. Proteins sequences were subsequently scanned for domains with InterProScan^69^ and a predicted protein model was built using the Phyre2 web portal^70^. Signal peptide, O- and N-glycosylation sites were predicted based on amino acid sequence by SignalP 5.0 server^71^, NetOGlyc 4.0 Server^72^ and NetNGlyc 1.0 server^73^, respectively. HTHMM v2.0 server^74^ was used to predict transmembrane helices and GPI-SOM to predict GPI-anchor^75^. MHC class I and II binding peptides were predicted using NetMHCpan-4.1^76^ and NetMHCIIpan-4.0^76^ and linear B cell epitopes with BepiPred Linear Epitope Prediction 2.0^77^.

### Expression and purification of recombinant proteins

Transcripts were cloned by overlapping PCR from previously designed artificial genes and cloned as NcoI-SalI fragments into the pHIS-parallel 2 expression vector^78^. For Gene 1 forward primer (FW) CGCCATGGGAGACGATTGCAGAAACGGAACTAGA and reverse primer (RV) CGGTCGACTAGTACGTTTTCCCTTCCTTAATTATTTTCTGTG was used. For Gene 2 CGCCATGGGATCTACAAGTACTACTACCCATCCAGTG (FW) and CGGTCGACTACACCAAGGAAAAGTGCATATTCTCGTT (RV) and for Gene 13 CGCCATGGGACAGGTACCAGTGTTTCCCCCTGG (FW) and CGGTCGACTATTTCCTTGGCACGCAAATATGTCTG (RV) were used. Clones were induced with 1 mM Isopropyl-β-D-thiogalactoside (IPTG) for 16 h at 20 °C in E. coli BL21 C41(DE3). The bacterial cells were then lysed and centrifuged. The expressed insoluble proteins were extracted from the inclusion bodies with the following protocol. The pellets were thoroughly homogenized in Phosphate Buffered Saline (PBS); 2% Triton X-100 followed by an incubation at 37 °C for 30 min with shaking. The samples were ultracentrifuged at 96,000 g for 30 min and the pellets were homogenized again in PBS and incubated at 37 °C for 30 min with shaking. After a second ultracentrifugation, the pellets were homogenized in PBS; 7 M urea. The denatured proteins were dialyzed to 2 M urea overnight.

### Preliminary RNA interference study

Silencing of the gene candidates by RNA interference (Genes 1, 2, and 13) was done as described previously (1). The Borrelia-infected nymphs were injected with 0.32 nl of dsRNA, rested for three days, and fed on C3H mice (5 nymphs per mouse, infection rate of ticks > 90%). The level of silencing was checked by qRT-PCR on a mix of five fully-fed nymphs per group and compared to the GFP control; expression of gene 3 was reduced by 92%, expression of gene 2 was reduced by 98%, expression of gene 13 was reduced by 99% and for gene 1 expression was reduced by 68% (primers can be found in Supplemental Table 1). The mice were screened for infection by qRT-PCR in a skin, heart, and urinary bladder, as described below.

### Vaccination-transmission studies and infection parameters

Pathogen-free C3H/HeN mice (Charles River Laboratories) were used for the vaccination transmission experiments. Six mice per group were vaccinated with either PBS, recombinant Gene 1, Gene 2, Gene 13 or all three recombinant proteins injected subcutaneously at different sites. 20 µg of antigen was emulsified in Complete Freund's Adjuvant (Sigma-Aldrich, St. Louis, MO, USA) to 100 µl total volume for prime vaccination at day 0. For booster vaccinations at day 14 and 28, 20 µg of antigen were emulsified in Incomplete Freund's Adjuvant, 100 µl total volume. 2 weeks after the last vaccination, mice were challenged with 5 *B. afzelii-*infected *I. ricinus* nymphs (infection rate > 90%) which were allowed to feed to repletion. Before each vaccination and the tick challenge, mouse blood was collected. 3 weeks after tick infestation, mice were sacrificed and organs were collected for culture and qPCR. Half of the mouse bladder and a part of the tick bite site were cultured in BSK medium (Amsterdam UMC, AMC, The Netherlands).

Total spirochete load in mouse tissues was determined by qPCR, which targeted a fragment of the *flagellin* gene (154 bp). DNA was isolated from individual murine tissues (ear, skin, heart, and urinary bladder) using a NucleoSpin tissue kit (Macherey–Nagel) according to the manufacturer’s protocol. The reaction mixture contained 12.5 μl of FastStart universal probe master (Rox) (Roche), 10 pmol of primers FlaF1A and FlaR1, 5 pmol of TaqMan probe Fla Probe1^8,8^, 5 μl of DNA, and PCR water up to 25 μl. Quantification of murine β-actin was performed using MmAct-F and MmAct-R primers and a MmAct-P TaqMan probe^14^. The following amplification program was run on a LightCycler 480 (Roche) for both targets: 95 °C for 10 min, 50 cycles at 95 °C for 15 s and 60 °C for 1 min. The spirochete burden in murine tissues was expressed as the number of spirochetes per 10^5^ murine β-actin copies.

### Antibody responses

Total antigen-specific IgG levels were determined by ELISA. ELISA plates (Thermo Scientific) were coated with full length proteins at 0.05 μg/well in carbonate buffer (pH 9.6) and incubated overnight at 4 °C. After washing with PBS, containing 0.05% Tween 20, the plates were incubated with blocking buffer (10% of fetal calf serum (FCS, Biowest) in PBS) for 1 h. Mouse sera were added at 1:5600 dilution and incubated for 2 h at room temperature. After washing, goat anti-mouse total IgG conjugated to horseradish peroxidase (HRP) (Jackson ImmunoResearch) was added (1:1000 dilution) in blocking buffer and incubated for 1 h at room temperature. The plates were then extensively washed and incubated with KPL SureBlue substrate. The reaction was stopped with 2N H_2_SO_4_. Absorbance (450 nm) was immediately measured using a BioTek Synergy HT multi-detection microplate reader.

## Supplementary information

Supplementary Figures.

Supplementary Information.
