# Supplementary material for: A combined transcriptomic approach to identify candidates for an anti-tick vaccine blocking B. afzelii transmission
Source: Sci Rep. 2020 Nov 18;10:20061. doi: 10.1038/s41598-020-76268-y (PMC7674437; doi:10.1038/s41598-020-76268-y)
Supplement: Supplementary file 1 — Supplementary Figures. [file 41598_2020_76268_MOESM1_ESM.pptx]

## Slide 1
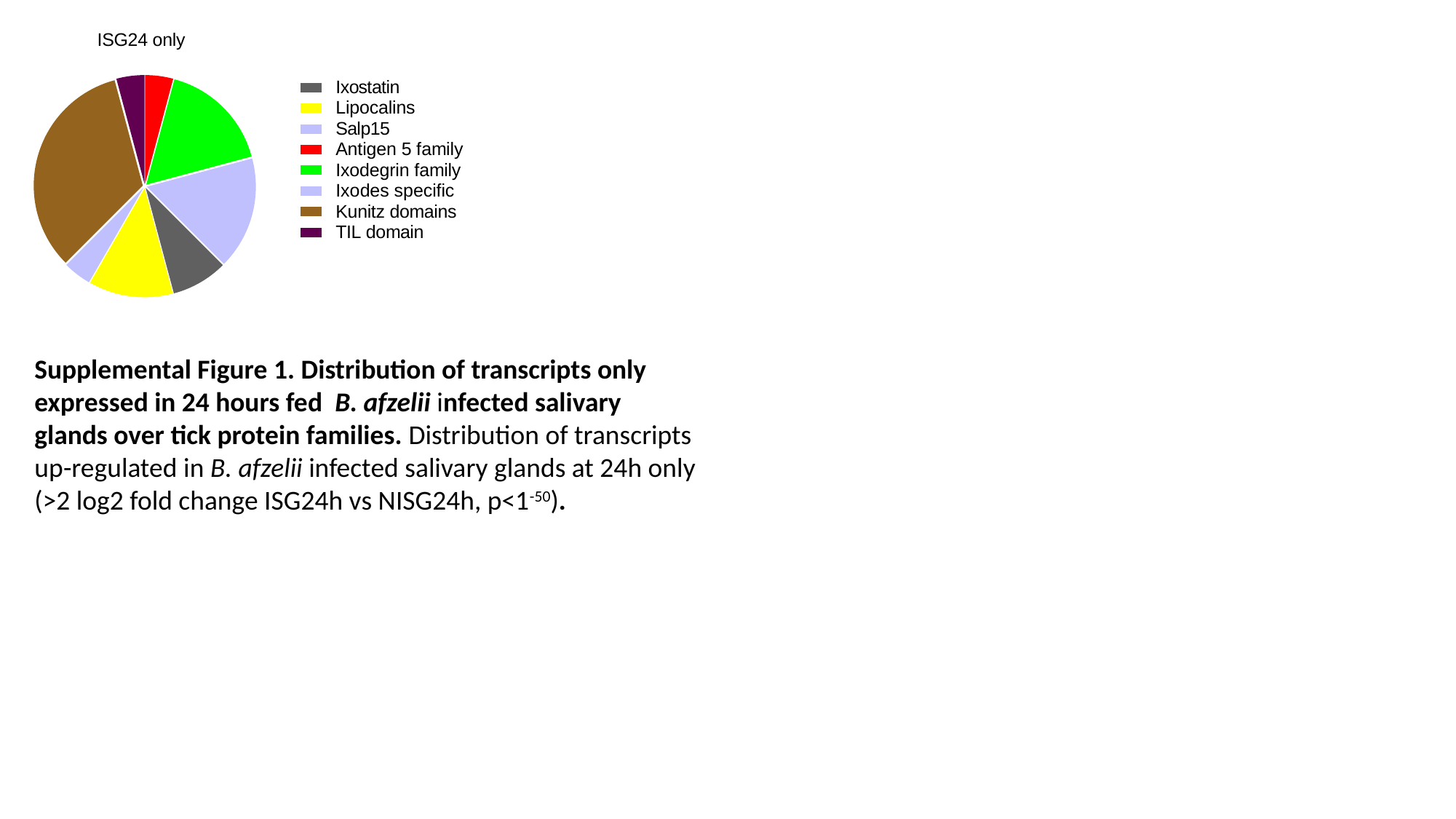

Supplemental Figure 1. Distribution of transcripts only expressed in 24 hours fed B. afzelii infected salivary glands over tick protein families. Distribution of transcripts up-regulated in B. afzelii infected salivary glands at 24h only (>2 log2 fold change ISG24h vs NISG24h, p<1-50).

## Slide 2
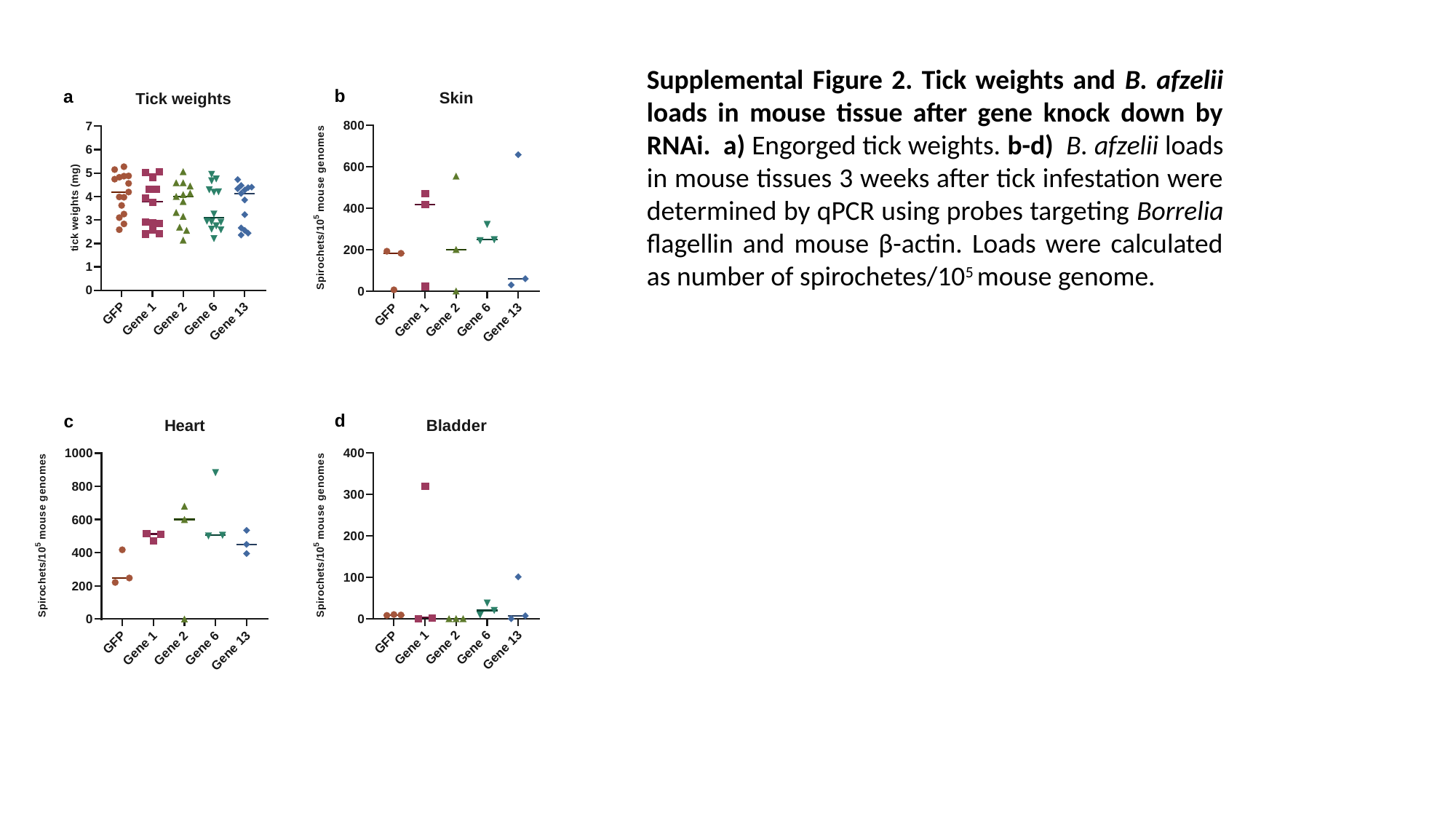

Supplemental Figure 2. Tick weights and B. afzelii loads in mouse tissue after gene knock down by RNAi. a) Engorged tick weights. b-d) B. afzelii loads in mouse tissues 3 weeks after tick infestation were determined by qPCR using probes targeting Borrelia flagellin and mouse β-actin. Loads were calculated as number of spirochetes/105 mouse genome.

## Slide 3
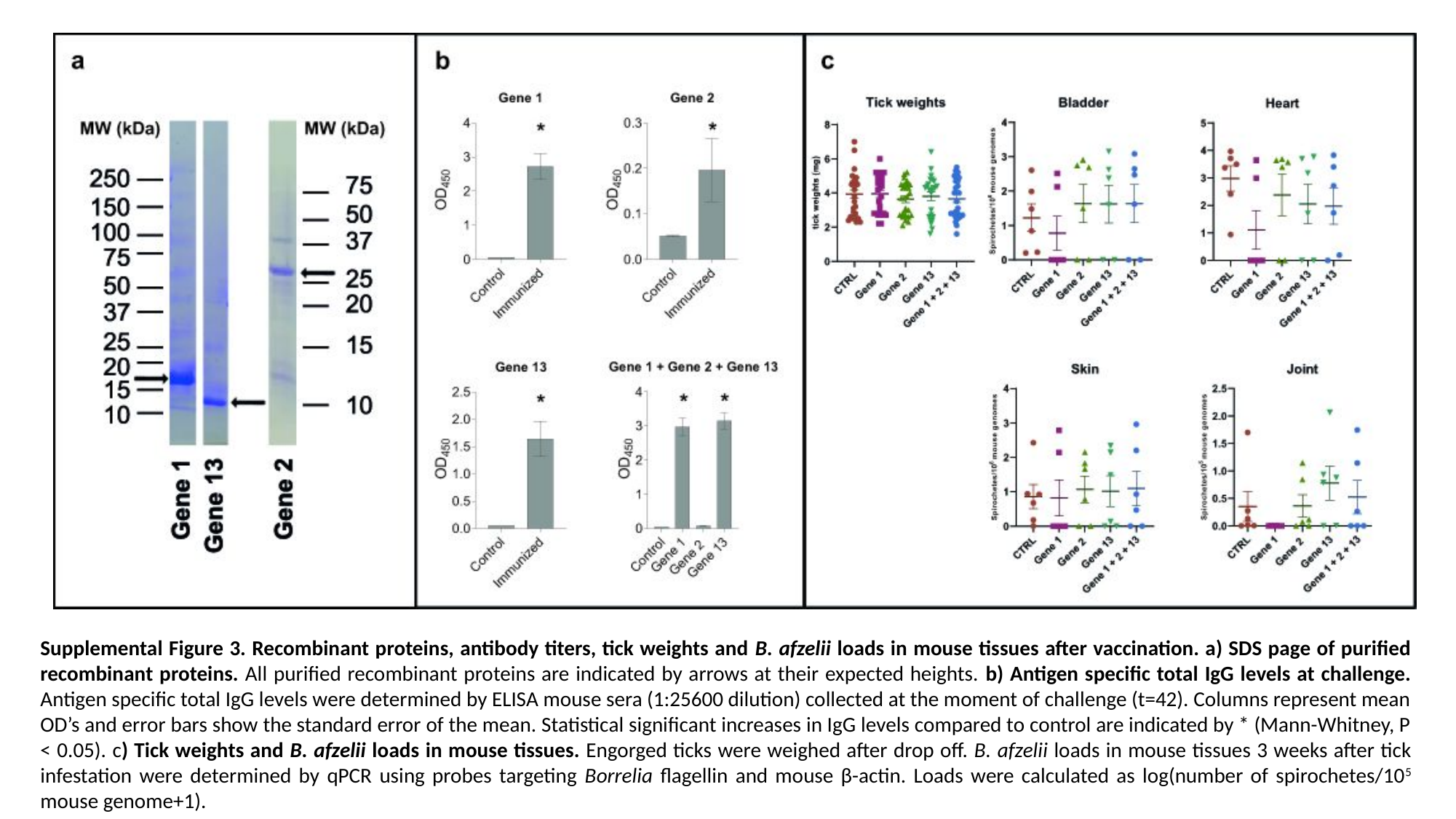

Supplemental Figure 3. Recombinant proteins, antibody titers, tick weights and B. afzelii loads in mouse tissues after vaccination. a) SDS page of purified recombinant proteins. All purified recombinant proteins are indicated by arrows at their expected heights. b) Antigen specific total IgG levels at challenge. Antigen specific total IgG levels were determined by ELISA mouse sera (1:25600 dilution) collected at the moment of challenge (t=42). Columns represent mean OD’s and error bars show the standard error of the mean. Statistical significant increases in IgG levels compared to control are indicated by * (Mann-Whitney, P < 0.05). c) Tick weights and B. afzelii loads in mouse tissues. Engorged ticks were weighed after drop off. B. afzelii loads in mouse tissues 3 weeks after tick infestation were determined by qPCR using probes targeting Borrelia flagellin and mouse β-actin. Loads were calculated as log(number of spirochetes/105 mouse genome+1).

## Slide 4
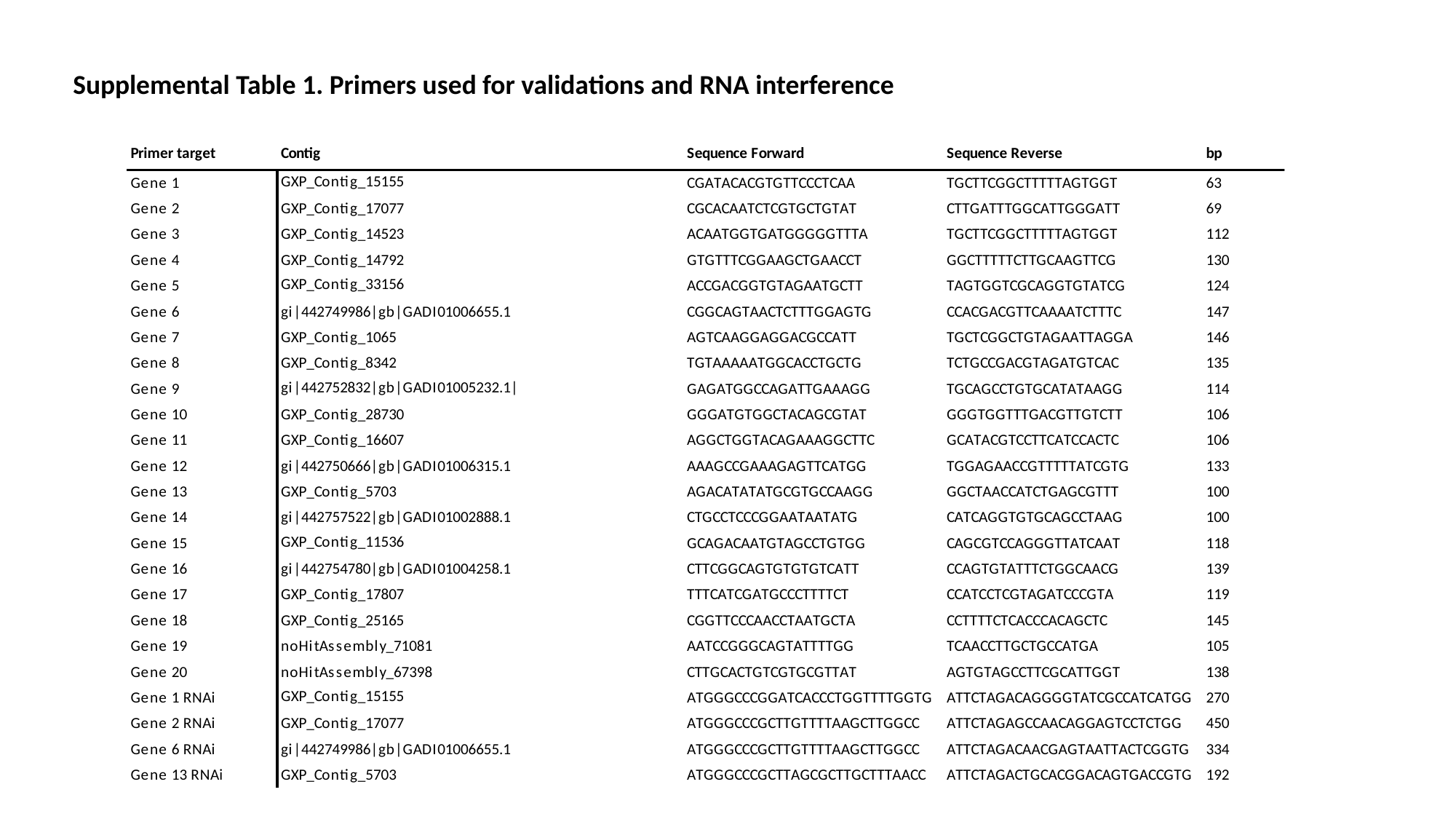

Supplemental Table 1. Primers used for validations and RNA interference

## Slide 5
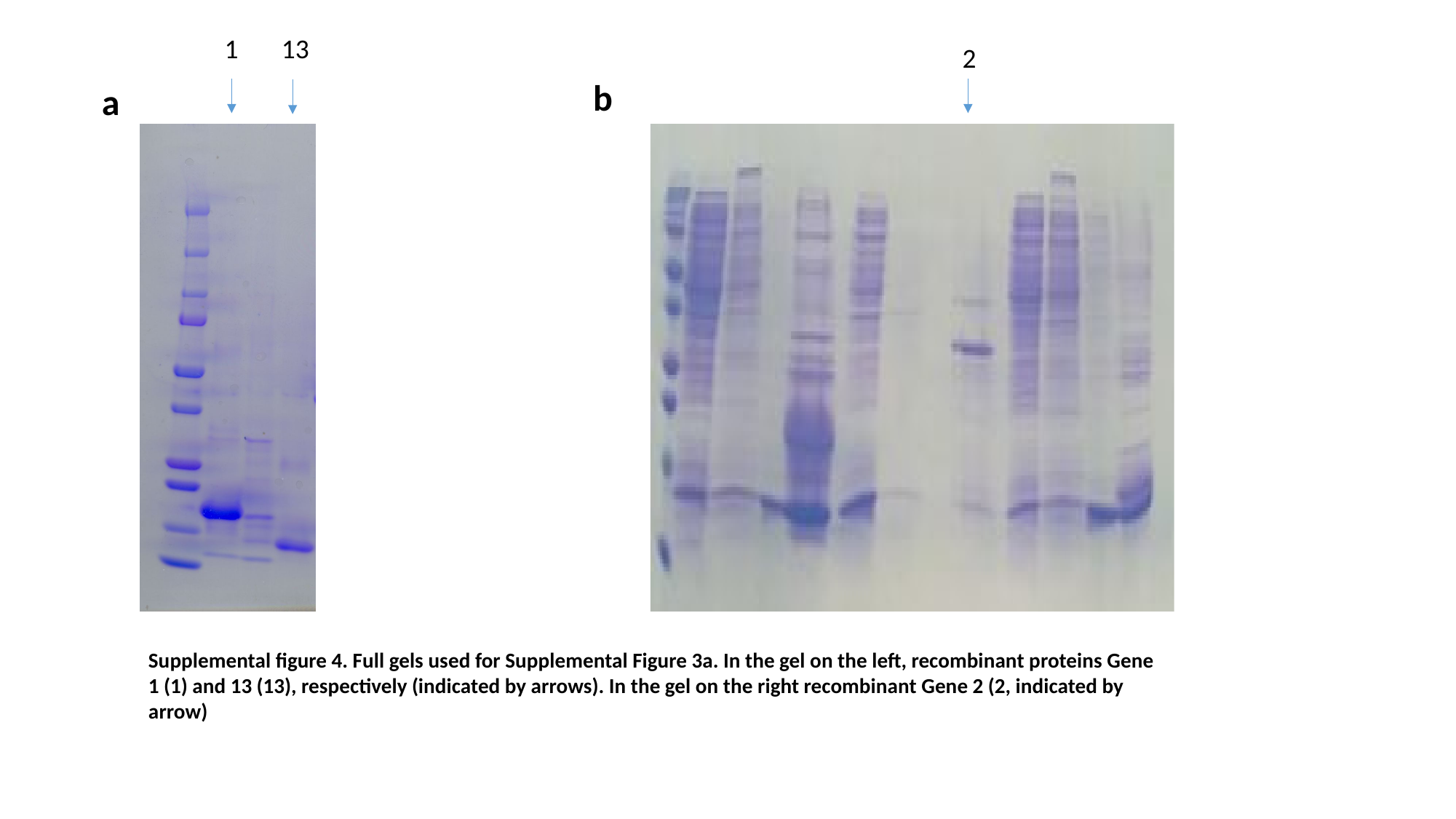

13
1
2
b
a
Supplemental figure 4. Full gels used for Supplemental Figure 3a. In the gel on the left, recombinant proteins Gene 1 (1) and 13 (13), respectively (indicated by arrows). In the gel on the right recombinant Gene 2 (2, indicated by arrow)
